# Coating Ti6Al4V implants with nanocrystalline diamond functionalized with BMP-7 promotes extracellular matrix mineralization in vitro and faster osseointegration in vivo

**DOI:** 10.1038/s41598-022-09183-z

**Published:** 2022-03-28

**Authors:** Ivana Nemcakova, Andrej Litvinec, Vaclav Mandys, Stepan Potocky, Martin Plencner, Martina Doubkova, Ondrej Nanka, Veronika Olejnickova, Barbora Sankova, Martin Bartos, Egor Ukraintsev, Oleg Babčenko, Lucie Bacakova, Alexander Kromka, Bohuslav Rezek, David Sedmera

**Affiliations:** 1 Institute of Physiology of the Czech Academy of Sciences, Videnska 1083, 142 20 Prague 4, Czech Republic; 2 Department of Pathology, Charles University, Third Faculty of Medicine, Ruska 2411, 100 00 Prague 10, Czech Republic; 3 Institute of Physics, Czech Academy of Sciences, Cukrovarnicka 10, 162 00 Prague 6, Czech Republic; 4 Institute of Anatomy, Charles University, First Faculty of Medicine, U Nemocnice 3, 128 00 Prague 2, Czech Republic; 5 Institute of Dental Medicine, Charles University, First Faculty of Medicine, U Nemocnice 2, 1280 00 Prague 2, Czech Republic; 6 Faculty of Electrical Engineering, Czech Technical University in Prague, Technicka 2, 166 27 Prague 6, Czech Republic

**Keywords:** Cell biology, Medical research, Materials science

## Abstract

The present study investigates the effect of an oxidized nanocrystalline diamond (O-NCD) coating functionalized with bone morphogenetic protein 7 (BMP-7) on human osteoblast maturation and extracellular matrix mineralization in vitro and on new bone formation in vivo. The chemical structure and the morphology of the NCD coating and the adhesion, thickness and morphology of the superimposed BMP-7 layer have also been assessed. The material analysis proved synthesis of a conformal diamond coating with a fine nanostructured morphology on the Ti6Al4V samples. The homogeneous nanostructured layer of BMP-7 on the NCD coating created by a physisorption method was confirmed by AFM. The osteogenic maturation of hFOB 1.19 cells in vitro was only slightly enhanced by the O-NCD coating alone without any increase in the mineralization of the matrix. Functionalization of the coating with BMP-7 resulted in more pronounced cell osteogenic maturation and increased extracellular matrix mineralization. Similar results were obtained in vivo from micro-CT and histological analyses of rabbit distal femurs with screws implanted for 4 or 12 weeks. While the O-NCD-coated implants alone promoted greater thickness of newly-formed bone in direct contact with the implant surface than the bare material, a further increase was induced by BMP-7. It can be therefore concluded that O-NCD coating functionalized with BMP-7 is a promising surface modification of metallic bone implants in order to improve their osseointegration.

## Introduction

Metallic alloys are the most widely used materials for the fabrication of load-bearing orthopaedic and dental implants that require outstanding mechanical properties such as excellent ductility, fatigue life and strength^1–3^. Titanium-based Ti6Al4V alloy possesses significantly lower density and stiffness (elastic modulus), matching the properties of cortical bone much more closely than stainless steel or Co-Cr-based alloys^2,3^. Moreover, Ti6Al4V has superior biocompatibility, the highest rate of integration with the host bone and the greatest corrosion resistance of these three implant alloys, thanks to the spontaneous formation of an adhesive titanium dioxide layer at its surface with the highest rate of spontaneous recovery after mechanical disruption^2,4,5^. Although Ti6Al4V is one of the most favourable metallic implant materials, many patients have experienced increasing local pain, swelling, allergic reactions, and inflammation resulting in implant migration, aseptic loosening, and osteolysis. These reactions increase the need for painful and expensive revision surgeries. Human life expectancy has been increasing, but a recent study has concluded that only about half of hip replacements last for 25 years in patients with osteoarthritis^6^. Many patients nowadays outlive their implant. There is therefore great interest in the innovation and optimization of promising biomaterials such as Ti6Al4V with a view to eliminating factors responsible for implant failure.

The most common causes of implant failure are an accumulation of wear debris (i.e. metallic ions from Ti6Al4V), insufficient implant integration into the host bone (osseointegration), or a bacterial infection^7^. Various types of functional coatings for metallic implants, such as hydroxyapatite-based coatings^8,9^, ceramic-based coatings^10,11^, carbon-based coatings, such as diamond-like carbon (DLC)^12,13^ or fullerene coatings^14,15^, and silver-containing nanocomposite coatings^16^ are therefore currently under investigation to address some or all of these issues. Another promising material for coating metallic implants, due to its superior properties, is a nanocrystalline diamond (NCD) coating. The main advantages of an NCD coating over other carbon-based coatings are that NCDs are optically transparent, chemically inert, highly biocompatible, and can easily be functionalized in many ways according to their intended application^17^. In addition, NCDs exhibit excellent mechanical hardness as well as high chemical, thermal and wear resistance^18,19^, which should limit their decomposition or transformation into materials with potential toxicity and accumulation of wear debris near the implant site. A publication by Papo et al*.* has indeed shown that an NCD coating markedly improved the wear resistance of Ti6Al4V alloy after simulated loading mimicking two years of clinical use^20^. Moreover, NCD-coated metallic implants should also lower the risk of post-surgical problems associated with a bacterial infection. A considerable number of studies have demonstrated strong antibacterial activity of NCD coatings against various Gram-negative and Gram-positive bacteria, including *Escherichia coli*, *Pseudomonas aeruginosa* and *Staphylococcus aureus*^21–24^.

In addition, the nanostructured morphology of NCD coatings better imitates the nanoscale architecture of natural bone, and this should promote better osseointegration of an implant into the surrounding bone tissues of a patient. Several studies have shown that NCDs deposited on various substrates, or diamond nanocrystallites added into hydrogels, stimulated osteogenic maturation (evaluated by increased expression of osteogenic markers, e.g., osteocalcin, collagen type I, increased activity of alkaline phosphatase, or increased mineralization of extracellular matrix) of various bone-derived cells in vitro^25–29^. Usually, however, not all osteogenic markers were elevated but only some of them, depending on the type of cell that was used, or depending on the form of the NCD material that was used. Although these in vitro data are quite promising, in vivo studies evaluating the osseointegration of diamond-coated implants are quite scarce and the results are ambiguous. While Kloss et al. reported enhanced new bone formation promoted by Ti6Al4V implants coated with oxygen terminated NCD coatings^30^, and Jaatinen et al*.* reported enhanced new bone formation promoted by Ti implants coated with amorphous diamond coatings^31^, studies by Rupprecht et al*.* and by Metzler et al*.* have failed to show a positive effect of NCD coatings on the osseointegration of Ti6Al4V implants in vivo^32,33^.

Improvements to the early stages of healing after an implant placement are very important because they can greatly enhance the clinical outcome. Implants that are osseointegrated more rapidly can be loaded sooner, and this is increasingly favoured by patients^34^. A possible way to improve the osteoconductive properties of NCD-coated implants in vivo is by functionalizing NCD coatings with a biomolecule inducing bone formation and regeneration.

Bone morphogenetic protein 7 (BMP-7), also known as osteogenic protein-1, has been recognized as a key modulator of bone and cartilage formation and repair, mainly through the SMAD pathway^35^. It has been reported that BMP-7 protein not only enhances osteogenic maturation of osteoblasts^36–38^ but also induces osteogenic differentiation of various stem cells^39–41^ and fibroblasts^42,43^ in vitro. Moreover, various animal studies have proved the positive effect of BMP-7-coated implants^43–45^, or of an administration of BMP-7 into an implant site^47,48^, or into a wound site without the use of any implant^49–52^ on the regeneration and formation of new bone in vivo. Because of its great osteoinductive effect, BMP-7 protein has been approved by the FDA for clinical application in the healing of fracture non-unions^53,54^.

The present study set out to assess the benefits of the NCD surface functionalization with BMP-7 protein in vitro and in vivo. The NCD coating deposited on Ti6Al4V alloy plates and screws used for experiments is modified by a plasma oxidation process to create an oxidized NCD (O-NCD) surface. The plasma oxidation process is known to clean a surface from organic residues and to provide a hydrophilic surface with various oxygen-related chemical groups, such as carboxyl and anhydride^55^. The oxidation process is therefore expected to improve both the physisorption of BMP-7 proteins and also the adhesion of human osteoblasts (compared to the hydrogenated surface), as has been observed in our previous studies^56,57^. The surface functionalization with BMP-7 has a potential to further enhance the osteogenic properties of the material both in vitro and in vivo. We therefore hypothesized that the surface modification of titanium alloy with O-NCD would increase its biocompatibility, manifesting as increased cell adherence, and faster osteogenic cell differentiation, and that these properties would be further enhanced by functionalization with BMP-7. To assess the benefits of this modification, firstly the effect of O-NCD surface with or without BMP-7 coating on phenotypic maturation of osteoblasts and on mineralization of the extracellular matrix is evaluated in vitro. Secondly, the effect of these coatings on osseointegration in vivo is evaluated by micro-CT and histology analyses after implantation into the femurs of New Zealand white rabbits for a period of 4 or 12 weeks. Standard material and morphological analyses of NCD coating and BMP-7 adhesion, thickness and morphology are also included.

## Materials and methods

### Materials

A conventional Ti implant alloy (Ti6Al4V) was used for the experiments in the form of thin plates (10 × 10 × 3 mm; BEZNOSKA Ltd., Czech Republic) and in the form of self-cutting screws (HA 3.5 × 8 mm; Medin Inc., Czech Republic).

### Sample preparation and characterization—nanocrystalline diamond coating

The Ti6Al4V plates and screws were coated with a thin nanocrystalline diamond (NCD) film. The samples were first pre-treated in a suspension of ultra-dispersed detonation nanodiamonds (DND, nominal size of 5 nm; New Metals and Chemicals Corporation Ltd., Japan). The diamond thin film growth was performed in the low-temperature low-pressure linear antenna microwave plasma (LAMWP) chemical vapour deposition (CVD) system (AK 400, Roth & Rau, Germany)^58^ for 60 h at a total gas pressure of 10 Pa, microwave power of 2 × 1.7 kW and a gas mixture of 3.3% CH_4_ and 13.3% CO_2_ to H_2_. The substrate temperature was kept at 450 °C by resistive heating of the substrate holder. The deposition was repeated twice to overcoat the top and the bottom part of the screws. Surface hydrogenation of the sample was carried out in the LAMWP system by microwave hydrogen plasma. The surface of the diamond-coated samples was further modified by an oxidizing process using radio frequency oxygen plasma (100 W, 4 min). The resulting material coating was characterized by standard analytical methods: optical microscopy, scanning electron microscopy (SEM; Maia 3, TESCAN, Czech Republic) and Raman spectroscopy (WITec alpha300 RAS, Germany).

### Sample preparation and characterization—BMP-7 functionalization

The Ti6Al4V plates and screws with an NCD coating were sterilized in an autoclave (Tuttnauer Co. Ltd., Israel). A part of the samples coated with oxidized NCD was further functionalized with human bone morphogenetic protein 7 (BMP-7; BioLegend, Cat. No. 595602, USA) to promote phenotypic maturation of osteoblasts in vitro or to promote bone formation and thus to improve the osseointegration of the implants in vivo*.* The samples were functionalized with BMP-7 by incubating them in a BMP-7 solution with a concentration of 10 µg/mL in phosphate-buffered saline (PBS) for 24 h at room temperature (RT). The samples were subsequently washed in PBS (Sigma-Aldrich, Merck, Germany) and were immediately used for in vitro or in vivo experiments.

In order to study the adsorption of BMP-7 on the diamond surfaces and the interaction of BMP-7 with these coatings, an atomic force microscopy (AFM; Dimension ICON, Bruker, US) analysis was performed. For the AFM analysis, BMP-7 protein was deposited on flat monocrystalline diamond samples with hydrogenated (H-NCD) and oxidized (O-NCD) surfaces that were prepared by the same technology (microwave hydrogen plasma and radio-frequency oxygen plasma) as described above. BMP-7 solution droplets of 30 µL were applied on both samples for 24 h to allow the BMP-7 molecules to adsorb. Then the samples were rinsed with water and were dried by airflow. The thickness of the BMP-7 adsorbed layer was measured by the contact mode (CM) nanoshaving method^59^. Four 2 × 2 µm^2^ images were scanned in CM-AFM. Next, a 5 × 5 µm^2^ overscan was performed in PeakForce Quantitative Nanomechanical mode (PFQNM), which reveals details of the surface morphology and also differences in the adhesion and deformation of the surface layer after adsorption of BMP-7.

### In vitro experiments—phenotypic maturation of human osteoblasts hFOB 1.19

The human non-tumour osteoblast cell line (hFOB 1.19; ATCC CRL-11372™, USA) was used to evaluate the phenotypic maturation of osteoblasts cultured on the investigated materials (Ti6Al4V alloy plates coated with O-NCD films with or without BMP-7 functionalization; unmodified Ti6Al4V plates were used as a reference control material). hFOB 1.19 cells were induced to proliferate when cultured at a permissive temperature of 33.5 °C and were induced to differentiate into mature osteoblasts expressing the normal osteoblast phenotype when cultured at a restrictive temperature of 39.5 °C^60,61^. The samples, inserted into polystyrene 12-well tissue culture plates (well diameter 21 mm; TPP, Switzerland), were seeded with hFOB 1.19 cells and were cultured in a 1:1 mixture of Ham’s F12 medium and Dulbecco’s modified Eagle’s medium (Gibco, Cat. No. 11039, USA) supplemented with 10% foetal bovine serum (Sebak GmbH, Germany) and geneticin G418 (0.3 mg/mL; Gibco, USA) at 33.5 °C in a humidified air atmosphere containing 5% of CO_2_. When the cells reached confluence (3 days after seeding), the temperature was set to 39.5 °C (labelled as day 0 of differentiation) and the cells were cultured for an additional period of 21 days with the medium exchanged every 2–3 days. The metabolic activity of hFOB 1.19 cells cultured on the investigated materials was evaluated on days 0, 3, 10 and 21 of differentiation, while the phenotypic maturation (osteogenic differentiation) of the cells was assessed on days 3, 10 and 21. Three independent samples for each experimental group and time interval were used. The quantitative data were presented as the mean ± S.E.M. (Standard Error of the Mean).

*The metabolic activity* of the cells (the activity of the mitochondrial enzymes) was assessed by incubation of the samples in a final concentration of 40 µM Resazurin (Sigma-Aldrich, Merck, Germany) in the fresh complete cell culture medium mentioned above. After 1.5 h of incubation at 39.5 °C, the fluorescence of the solution was measured (Ex/Em = 530/590 nm) by a Synergy™ HT Multi-Mode Microplate reader (BioTek, USA). A solution without cells was used as a blank control.

*The activity of alkaline phosphatase* (ALP, a marker of early/mid-term osteogenic differentiation) was assessed on days 3, 10 and 21 of differentiation after 10-min incubation of samples in a solution of 1 mg/mL of *p*-nitrophenyl phosphate in a substrate buffer (50 mM glycine, 1 mM MgCl_2_, pH 10.5; Sigma-Aldrich, Merck, Germany) at RT. The resulting solution was then mixed with the same volume of 1 M NaOH solution, and the absorbance was measured at 405 nm by a Synergy™ HT Multi-Mode Microplate reader (BioTek, USA). A solution without cells was used as a blank control. The absorbance of the known concentrations of *p*-nitrophenol diluted in 0.02 M NaOH (9–90 μM; Sigma-Aldrich, Merck, Germany) was measured as a standard.

*The extracellular matrix mineralization* (a late marker of osteogenic differentiation) was evaluated on days 10 and 21 of differentiation by Alizarin Red S staining. The cells cultured on the samples were fixed in 4% paraformaldehyde (PFA; Sigma-Aldrich, Merck, Germany) for 15 min at RT. The samples with fixed cells were incubated in 40 mM Alizarin Red S in dH_2_O (pH 4.1; Sigma-Aldrich, Merck, Germany) for 20 min at RT with gentle shaking. The unbound dye was washed away by dH_2_O (5 times for 10 min with gentle shaking) and the samples were then left to dry and were stored at -20 °C until the quantification was performed. To extract the dye, the samples were incubated in 10% acetic acid for 1 h at RT with gentle shaking. The solution that was obtained, together with the layer of cells (detached by a cell scraper), was transferred to a 1.5 mL tube, was vortexed for 30 s and was heated at 85 °C for 10 min. The tubes were then incubated on ice for 10 min and were subsequently centrifuged at 20,000*g* for 15 min. The pH of the supernatant was adjusted by 10% NH_4_OH (Sigma-Aldrich, Merck, Germany) to 4.2, and the absorbance of the solution was measured at 405 nm by a Synergy™ HT Multi-Mode Microplate reader (BioTek, USA). A solution without cells was used as a blank control.

*The real-time qPCR method* was used to evaluate the expression of other markers of phenotypic maturation of hFOB 1.19 cells cultured on the investigated materials. RNA from the cells was extracted using a Total RNA Purification Plus Micro Kit (Norgen BioTek, USA), according to the manufacturer’s instructions. An amount of 300 ng/µL mRNA was used for reverse transcription into cDNA using the ProtoScript First Strand cDNA Synthesis Kit (New England Biolabs, USA) with oligo-dT primers. The reaction ran in a T-Personal Thermocycler (Biometra GmbH, Germany). The relative mRNA expression was quantified using SYBR Green (FastStart Universal SYBR Green Master; Roche Diagnostics GmbH, Switzerland) and primers from Generi Biotech, as presented in Table 1. The cDNA was amplified in the iCycler iQ™ 5 Multicolor Real-Time PCR detection system (BioRad, USA) in a total reaction volume of 20 μl, under the following conditions: 10 min incubation at 95 °C, followed by 40 cycles of 15 s at 95 °C and 1 min at 60 °C. The data were analysed using the ${2}^{{-\Delta \Delta C}_{t}}$ method, were normalized against the GAPDH housekeeping gene, and are plotted as mean ± standard deviation (SD).

**Table 1:** Oligonucleotide primers used for qPCR amplification.

Gene	Forward primer sequence	Reverse primer sequence	Product length (bp)
BGN	5′-CAG CCC GCC AAC TAG TCA-3'	5′-GGC CAG CAG AGA CAC GAG-3'	93
COL1A1	5′-CAG CCG CTT CAC CTA CAG C-3′	5′-TTT TGT ATT CAA TCA CTG TCT TGC C-3′	83
DCN	5′-GGA GAC TTT AAG AAC CTG AAG AAC C-3'	5′-CGT TCC AAC TTC ACC AAA GG-3'	104
GAPDH	5′-TGC ACC ACC AAC TGC TTA GC-3'	5′-GGC ATG GAC TGT GGT CAT GAG-3'	87
OSX	5′-GGC ACA AAG AAG CCG TAG TC-3'	5′-CAG GTG AAA GGA GCC CAT TA-3'	106
RUNX-2	5′-GCC TTC AAG GTG GTA GCC C-3'	5′-CGT TAC CCG CCA TGA CAG TA-3'	67
SPARC	5′-GTA CAT CGC CCT GGA TGA GT-3'	5′-CGA AGG GGA GGG TTA AAG AG-3'	124

*BGN* biglycan, *COL1A1* collagen type I, *DCN *decorin, *GAPDH* glyceraldehyde 3-phosphate dehydrogenase, *OSX* osterix, *RUNX-2* runt-related transcription factor 2, *SPARC* osteonectin.

*A statistical analysis* was performed using SigmaStat (Jandel Corporation, USA). A comparison between the groups was analysed with the ANOVA, Student–Newman–Keuls method. Values of p < 0.05 were considered statistically significant.

### In vivo experiments on rabbits

The animal experiments were approved by the Animal Care and Use committee of the Institute of Physiology in compliance with the current national legislation. The experiments conformed with all the international and EU ethical standards and also with the ARRIVE guidelines (https://arriveguidelines.org/arrive-guidelines).

New Zealand white rabbits were supplied by an accredited supplier (Velaz, Prague, Czech Republic) and were provided with food and water ad libitum. Animals of either sex were used (total N: 44), with a minimum body weight of 3.4 kg. After a habituation period of at least one week, the animals were weighed, and their body weights were recorded. Anaesthesia was initiated by an intramuscular (IM) injection of diazepam (5 mg/kg). The surgery was performed under antibiotic cover of marbofloxacin (5 mg/kg IM). Deep anaesthesia (surgical plane) was induced by ketamine (50 mg/kg, IM) with xylazine (5 mg/kg, IM). After the cessation of reaction to painful stimuli, a dab of antiseptic ointment (Ophtalmo-Septonex) was applied to both eyes to prevent corneal drying, and the limb to be operated on was shaved from ankle to groin. The surgical field was then thoroughly disinfected by povidone iodine and the animal was placed onto an operating table, where the surgical plane of anaesthesia was maintained by continuous administration of 2% halothane (Narcotan) with oxygen applied via a face mask. Pulse and blood oxygen saturation were continuously monitored by a pulse oximeter.

After the field had been covered with sterile wraps, a skin incision was performed in the area of the lateral condyle of the femur, followed by blunt dissection of all the layers up to the bone. After finding and marking the appropriate place on the lateral aspect, a hole 2.7 mm in diameter was drilled by an orthopaedic drill (Acculan 3Ti, B.Braun), followed by the implantation of O-NCD-coated, O-NCD + BMP-7-coated or uncoated control Ti6Al4V screws (HA 3.5 × 8 mm) via a hand screwdriver. To reduce the necessary number of animals (3R), both sides were operated consecutively, i.e., 12 and 4 weeks before sampling, to obtain 2 samples at different time points from each animal. The minimum size for each group was 5 implants for each type of coating and sampling interval. The wound was then closed in layers with a PDS II 4–0 absorbable suture.

After the surgery, the animals received a depot dose of 0.5 mg/kg of non-steroidal anti-inflammatory analgesic meloxicam by a subcutaneous injection (SC), and the suture was smeared with Betadine to prevent infection of the wound. During the recovery period, the animals were monitored continuously until complete awakening. For at least 10 days after surgery, the animals were checked several times a day with emphasis on their general well-being and on wound healing. For a period of 5 days, a combination of an antibiotic (marbofloxacin 5 mg/kg, IM) and an analgesic (meloxicam 0.5 mg/kg, IM) was administered daily. The diet was enriched with fresh vegetables and hay for faster healing. Food and water intake were monitored daily by a veterinarian for the entire duration of the experiment.

At the time of sampling, i.e., 4 or 12 weeks after implantation, the animals were sacrificed by an approved method (rendering them unconscious by a spring-loaded device followed by exsanguination), and the distal femurs containing the implants were extracted. The samples were sawed down to a size that fitted the fixation vials, and were subsequently fixed for 48 h in 4% paraformaldehyde in PBS at 4 °C. After rinsing in PBS, the samples were scanned and evaluated by micro-CT (see 2.6), as in our previous studies^62,63^. After imaging, the samples were decalcified with a hydrochloric acid solution (Histolab Products, Sweden) and the screws were carefully removed after slicing the decalcified bone in half with a scalpel. The bone samples were then processed for paraffin histology (see below), while the screws were imaged using SEM to confirm the integrity of the coating.

### Micro-CT evaluation of the tissue samples

Each specimen was placed in a plastic tube with PBS and was scanned using a SkyScan 1272 micro-CT device (Bruker micro-CT, Kontich, Belgium) with the following scanning parameters: pixel size 10 μm, source voltage 100 kV, source current 100 μA, 0.11 mm Cu filter, frame averaging 3, rotation step 0.2°, rotation 360°. The scanning time was approximately 6 h for each specimen. The flat-field correction was updated before each acquisition. Image data were reconstructed and were processed using NRecon SW (Bruker, Belgium). Standardized cross-section images were acquired using DataViewer (Bruker, Belgium).

### Histological analysis of the tissue samples

PFA-fixed, paraffin-embedded tissue samples were cut in series at 10 µm and were stained alternately with Hematoxylin–Eosin and van Gieson/Orcein staining protocol. The stained sections were photographed using an Olympus DP80 CCD camera (Olympus, Tokyo, Japan) fitted on an upright BX51 compound Olympus microscope at different magnifications (2×–20×). The thickness of newly formed bone was measured in the mid-shaft region taking care to exclude any attached trabeculae. Digital images were processed using Adobe Photoshop (background and levels adjustment, Unsharp Mask filtering—always performed on the entire image) and were arranged in plates with the same software.

## Results and discussion

### Characterization of the NCD layer and BMP-7 adhered on NCD

**Figure 1:**
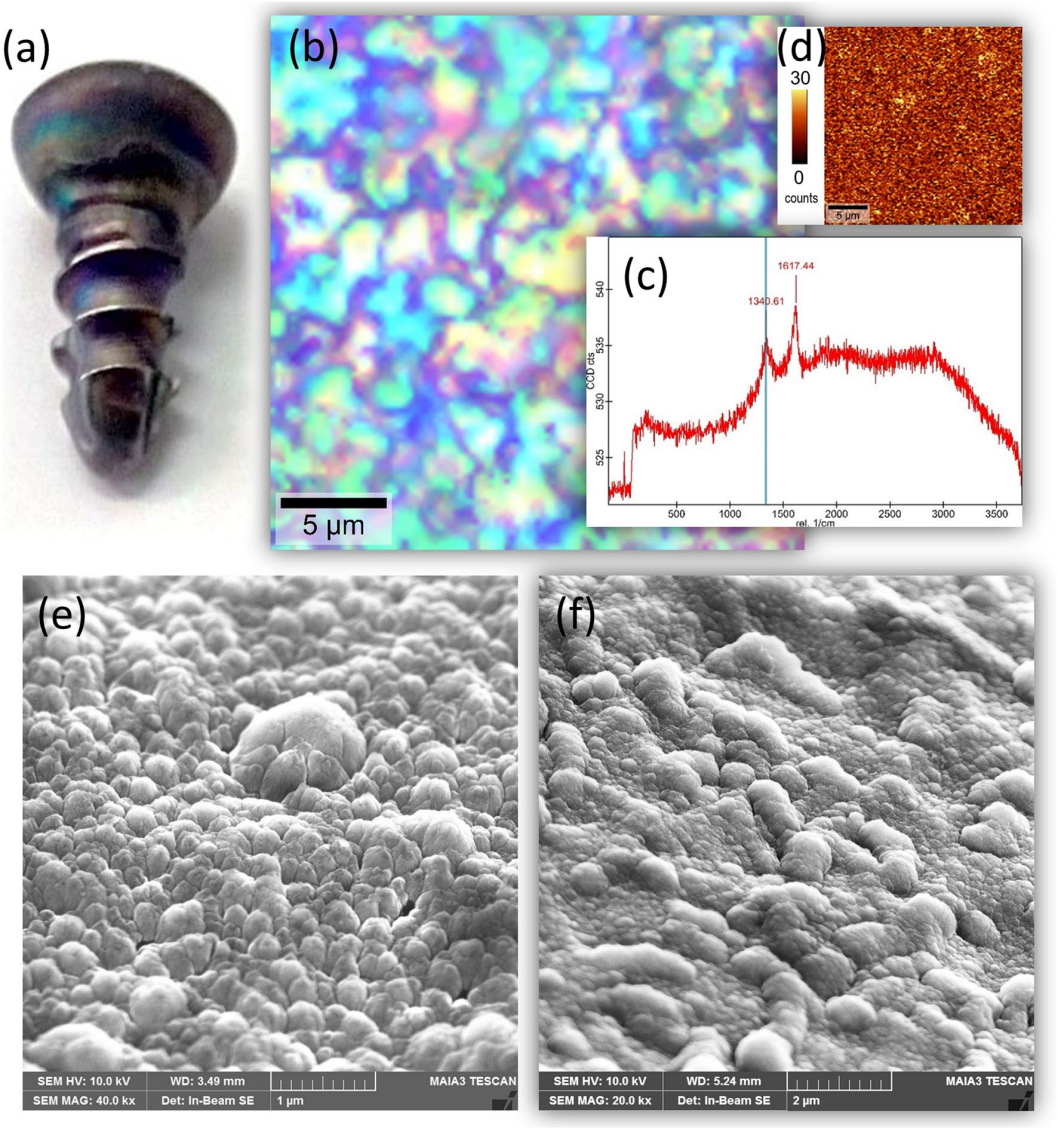
Material and morphological characteristics of the nanocrystalline diamond-coated cortical screws: **(a)** a photograph of a coated screw, **(b)** bright-field optical microscope image, **(c)** Raman spectrum showing characteristic nanocrystalline diamond features, **(d)** a Raman diamond peak intensity map, **(e)** SEM image of the coated screw surface along the thread and **(f)** in the screw tip area.

Raman spectroscopy and surface mapping show uniform coating by NCD with the typical diamond peak and G band in the spectrum (Fig. 1). The SEM image of the coated screw surface along the thread and in the screw tip area exhibits a corrugated morphology conformal to the screw surface finish and fine nanostructures related to the NCD grains in both critical areas.

**Figure 2:**
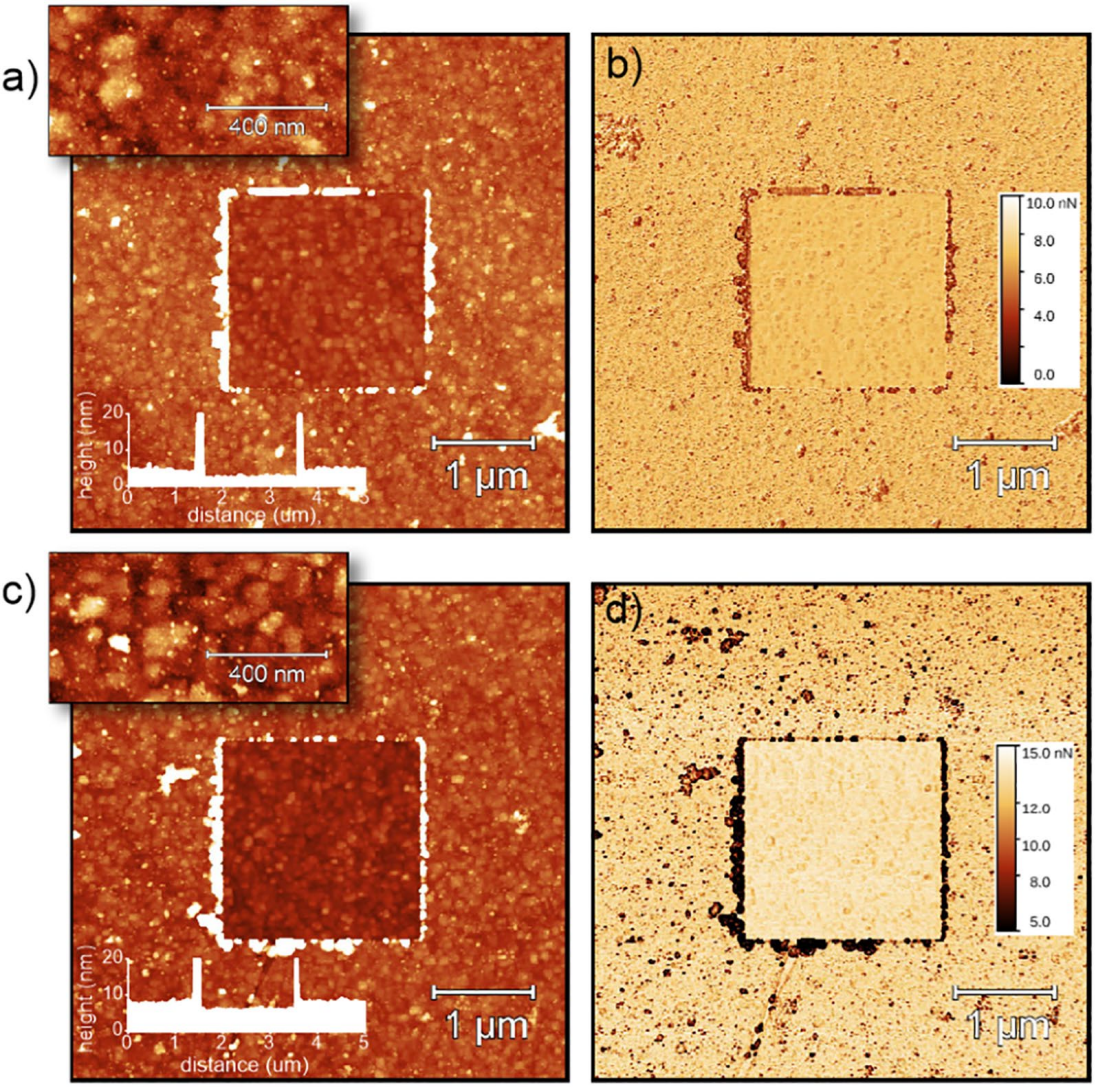
BMP-7 morphology (**a,c**), adhesion maps **(b,d)**, and thickness profiles (insets) observed by AFM in the PFQNM regime on oxidized (**a,b**) and hydrogenated (**c,d**) NCD surfaces. The square in the centre corresponds to the area from which the molecules were removed (nanoshaved) by scanning in contact mode AFM. The Z scale is 20 nm for 5 × 5 µm^2^ topography images, 10 nm for 0.5 × 1 µm^2^ topography images (insets) and 10 nN for 5 × 5 µm^2^ adhesion images.

The BMP-7 morphology, the adhesion map and the thickness profile observed by AFM in PFQNM regime O-NCD and H-NCD surfaces are shown in Fig. 2. The square in the centre corresponds to the area from which the molecules were removed (nanoshaved) by scanning on contact mode AFM. The BMP-7 adsorbed thickness on the O-NCD surface, as determined from the mean heights in the central and outside area, was 1.8 ± 1.3 nm. From the profile across the nanoshaved area, the thickness was also 1.8 ± 1.3 nm. The BMP-7 adsorbed thickness on the H-NCD surface was 1.9 ± 1.2 nm in area and 2.3 ± 1.2 nm from the line profile.

The thickness of the adsorbed BMP-7 was similar on the H-terminated and O-terminated nanocrystalline diamond surfaces. The BMP-7 surface morphology also appeared qualitatively similar, comprised of a uniform conformal coating and a high density of larger (brighter) dots. This is a noteworthy finding, as H-NCD is hydrophobic and O-NCD is hydrophilic. The adsorbed BMP-7 thickness was on average only 2 nm. The three-dimensional structure of BMP-7 exhibits an open boomerang-shaped conformation with dimensions of 13 × 9 × 5 nm^3^, as deduced from transmission electron microscopy (TEM) measurements and from Small Angle X-ray scattering (SAXS) measurements^64^. Thus, in our case, the BMP-7 molecules must be lying on the diamond surface, forming a uniform primary layer coating, on which the BMP-7 molecules, adsorbed in a more native form, correspond to the observed brighter dots. The relatively small and similar thickness of the BMP-7 primary layer on the hydrogenated and oxidized diamond surfaces may be explained by a tight interaction with the diamond surface dipoles (C-H or C-O) via hydrogen bonding. The BMP-7 could also be collapsed (as observed by comparing the AFM data in air and in solution on other biomolecules^57^. However, the BMP-7 dimensions given above were determined from the TEM in vacuum condition^64^, so this effect can be excluded.

However, the conformation of BMP-7 in the primary layer on H- and O-terminated NCD can differ, as was observed above for adsorbed FBS proteins^59,65^. This effect may be due to different polarity of the surface dipoles, different protein parts forming hydrogen bonds and/or interacting with the diamond surface via hydrophobic or hydrophilic interactions. This assertion is supported by the PFQNM adhesion data (tip-molecule adhesion forces in the range of 10 nN). There is a uniform background of the adhesion force due to the thin BMP-7 layer, which is lower than on the bare (nanoshaved) diamond in the central area of the adhesion images. The difference in the adhesion force between BMP-7 and bare diamond is greater on H-NCD than on O-NCD. This again hints at a slightly different conformation of the BMP-7 molecules lying on the surface. On both types of surfaces, there are also smaller and larger dark dots in the adhesion map, obviously corresponding to the native form of BMP-7. From the adhesion image, it is evident that these BMP-7 aggregates are larger and more pronounced on H-NCD, while they are fine and more homogeneously distributed on O-NCD. This again suggests that the BMP-7 interaction with H-NCD and O-NCD is different, despite the similar surface morphology and a similar thickness of approximately 2 nm.

### Phenotypic maturation of hFOB 1.19 cells in vitro

**Figure 3:**
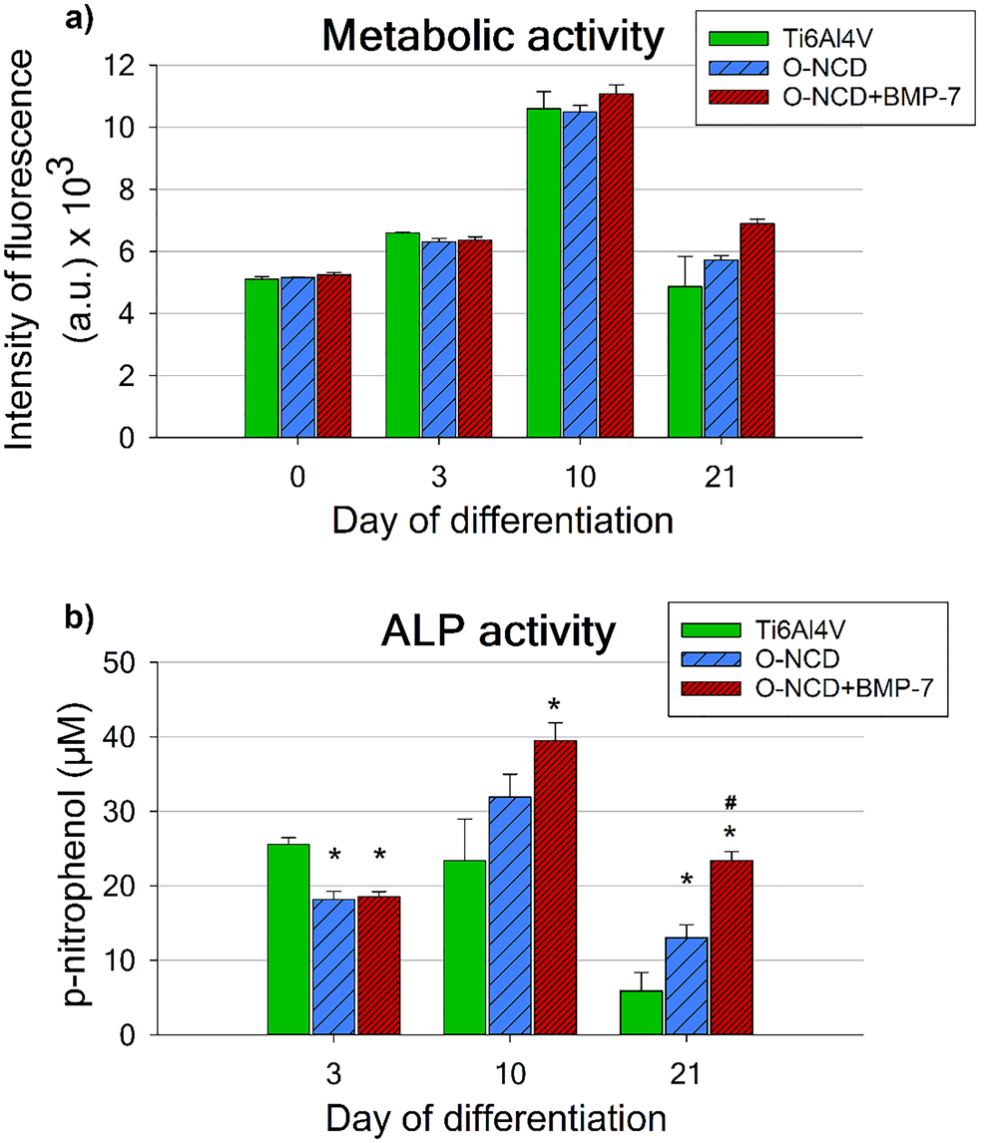
The metabolic activity **(a)** and the activity of alkaline phosphatase (ALP) **(b)** of hFOB 1.19 cells cultured on bare Ti6Al4V samples (Ti6Al4V), on Ti6Al4V samples coated with the O-terminated nanocrystalline diamond coating (O-NCD), or on Ti6Al4V samples coated with the O-terminated nanocrystalline diamond coating functionalized with BMP-7 (O-NCD + BMP-7) under differentiation conditions (a restrictive temperature of 39.5 °C) for 3 weeks. Mean ± S.E.M. * indicates a significant difference from the reference Ti6Al4V, while # shows a significant difference from the O-NCD coating; p < 0.05.

To investigate the effect of the O-NCD coating and the O-NCD coating functionalized with BMP-7 (O-NCD + BMP-7) on the maturation of human osteoblasts in vitro, confluent hFOB 1.19 cells were cultured for another 21 days at a restrictive temperature of 39.5°, inducing their differentiation into the mature osteoblast phenotype. Figure 3a shows that the values of the metabolic activity (which are usually proportional to the cell numbers) of hFOB 1.19 cells were comparable among all sample groups on days 0 and 3 of differentiation. The cells exhibited the greatest metabolic activity on day 10 of differentiation, with values 2-times higher than on the other evaluated days. Slightly higher metabolic activity of cells cultured on the O-NCD + BMP-7 coating was observed with increasing time of cultivation (from day 10 of differentiation); however, these differences were not proven to be significant (Fig. 3a). The results of metabolic activity at all assessed time intervals did not show any significant differences among the investigated materials.

**Figure 4:**
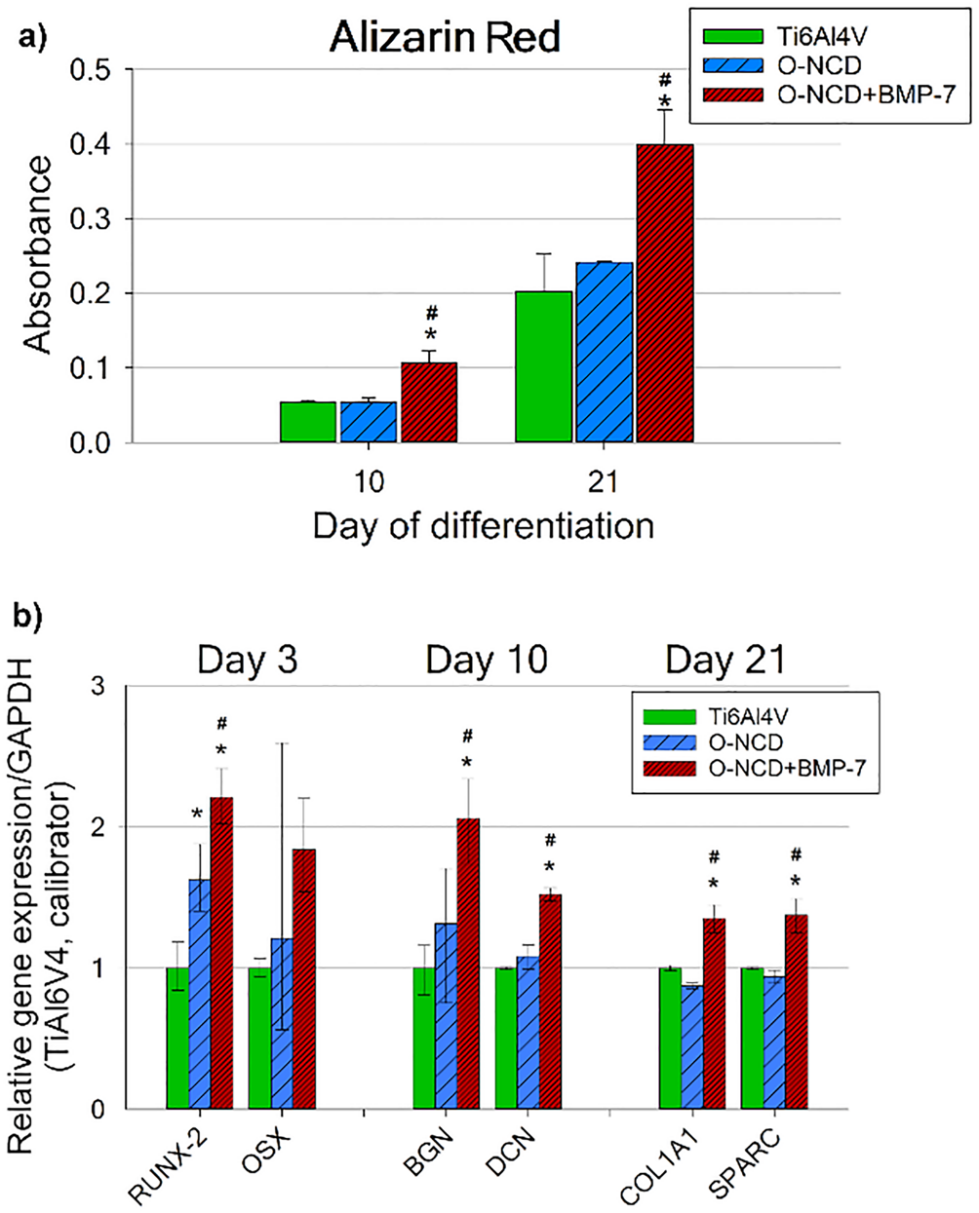
Mineralization of the extracellular matrix, assessed by Alizarin Red S staining, induced by hFOB 1.19 cells **(a**), and their relative gene expression** (b).** The cells were cultured on bare Ti6Al4V samples (Ti6Al4V), on Ti6Al4V samples coated with the O-terminated nanocrystalline diamond coating (O-NCD), or on Ti6Al4V samples coated with the O-terminated nanocrystalline diamond coating functionalized with BMP-7 (O-NCD + BMP-7) under differentiation conditions (a restrictive temperature of 39.5 °C) for 3 weeks. Graph **(b)** compares the gene expression of osteogenic markers RUNX-2 and OSX (osterix; both early markers), BGN and DCN (biglycan and decorin; mid-term markers) and COL1A1 with SPARC (collagen type I and osteonectin; late markers). The relative mRNA expression was quantified by the ${2}^{-\Delta \Delta Ct}$ method. The data are expressed relative to GAPDH, normalized to gene expression on the reference Ti6Al4V sample (calibrator). Mean ± S.E.M. **(a)**. Mean ± SD **(b)**. * indicates a significant difference from the reference Ti6Al4V, while # shows a significant difference from the O-NCD coating; p < 0.05.

The evaluation of the activity of alkaline phosphatase (ALP), which is one of the main markers of early/mid-term phenotypic maturation of osteoblasts, showed that on day 3 of differentiation, the activity of ALP in cells on both O-NCD-based coatings was lower than in cells on uncoated Ti6Al4V (Fig. 3b), while the gene expression of other early and mid-term markers of osteogenic differentiation, namely RUNX-2, osterix, biglycan and decorin, was usually increased (Fig. 4). However, it should be pointed out that the enzymatic activity cannot be always proportional to the expression of a given enzyme at mRNA and protein level, because enzymes, including ALP, can be present in their immature, pro-enzymatic form. The correlation between the gene expression of ALP, amount of its molecules and its activity in cells on O-NCD-based coatings should be further investigated.

A lower activity of ALP on O-terminated NCD surfaces might also be explained by a certain inhibitory effect of these surfaces on the enzymatic activity. On the one hand, O-NCD surfaces are hydrophilic, and thus they promote the adsorption of cell adhesion-mediating proteins in an appropriate geometric conformation for binding cell adhesion receptors, and by this mechanism, these surfaces support the adhesion, spreading and subsequent proliferation of cells. On the other hand, oxygenated surfaces can also have some adverse effects on cells. It is known that oxygen radicals can inhibit the activity of various enzymes, including ALP. For example, treatment of ALP with oxidizing agents, such as *p*-nitrophenyl phosphate, beta-glycerophosphate or ascorbic acid/Fe^2+^, resulted in the inhibition of the enzyme activity, due to generation of oxygen radicals, e.g., .OH radicals^66^. Other examples are inhibition of ALP by H_2_O_2_^67^ or low activity of serum ALP in patients with Wilson´s disease, where the enzyme is inhibited by ROS induced by elevated Cu^2+^ ions^68^. Oxygen-containing radicals might be present on the surface of NCD after oxygen plasma treatment, especially in relatively early culture intervals, when the cultivation substrate is not fully covered with cells and their newly formed extracellular matrix.

It is also known that ALP activity is inversely correlated with the concentration of calcium^69^. Ca^2+^ ions can be attracted to oxygen-containing chemical functional groups on O-NCD surface, which are usually negatively charged (-COOH^-^, OH^-^). Last but not least, as every protein, ALP can be adsorbed to the O-NCD surface, which can inhibit its activity. For example, ALP activity was deactivated by adsorption on quartz slides^70^.

Nevertheless, in the following culture intervals (days 10 and 21 of differentiation), the ALP activity of hFOB 1.19 cells cultured on the O-NCD-based coatings increased in comparison with the value on the uncoated Ti6Al4V, especially on O-NCD functionalized with BMP-7. The cells cultured on the O-NCD coating functionalized with BMP-7 revealed the highest ALP activity with significant differences from the reference Ti6Al4V alloy (days 10 and 21) and from the O-NCD coating (day 21). Nevertheless, after 21 days of differentiation, even the O-NCD coating without BMP-7 induced greater ALP activity of the cells than the reference Ti6Al4V. Similarly to the metabolic activity, the ALP activity of the hFOB1.19 cells was also about two times higher on day 10 than on days 3 and 21 (Fig. 3b).

The mineralization of the extracellular matrix, which is a typical marker of late osteogenic differentiation, was assessed by Alizarin Red S staining on days 10 and 21 of differentiation. The extracellular matrix mineralization by hFOB 1.19 cells was about 2 times higher on the O-NCD coatings functionalized with BMP-7 than on the reference Ti6Al4V and on O-NCD without BMP-7 at both evaluated time intervals (Fig. 4a).

The qPCR analysis performed on days 3, 10 and 21 of differentiation revealed that the gene expression of all monitored osteogenic markers (except for OSX) was significantly higher in cells growing on the O-NCD coatings functionalized with BMP-7 than on the other materials (Fig. 4b). The expression of both early osteogenic factors (RUNX-2, OSX), evaluated on day 3 of differentiation, showed a similar trend. The expression of RUNX-2 in cells cultured on O-NCD + BMP-7 was significantly higher than in cells grown on the reference Ti6Al4V and on O-NCD without BMP-7. This is in agreement with the fact that BMP proteins are known to stimulate RUNX-2 expression through the SMAD protein signalling pathway^34,71^. Moreover, even the O-NCD coating without BMP-7 induced a higher RUNX-2 expression than the reference Ti6Al4V. The expression of OSX, despite following a similar trend to RUNX-2, was not found to be significantly increased due to a big data spread. On day 10 of differentiation, the mid-term osteogenic markers BGN and DCN, which are associated with matrix mineralization in vitro^72,73^, also showed a significantly increased expression in cells cultured on O-NCD + BMP-7 in comparison with other materials. These results correspond with the matrix mineralization results on day 10 assessed by Alizarin Red S staining. The expression of late osteogenic markers, such as COL1A1 and SPARC^72^, measured on day 21 of differentiation, followed the same expression profile, with higher values found on the O-NCD-BMP-7 samples than on the reference Ti6Al4V and on the O-NCD coating without BMP-7 (Fig. 4b).

In summary, the osteogenic maturation of hFOB 1.19 cells in vitro was slightly enhanced by the O-NCD coating, which promoted a greater expression of an early osteogenic factor RUNX-2 and greater activity of alkaline phosphatase than the reference uncoated Ti6Al4V alloy. However, O-NCD coating of the Ti6Al4V alloy did not increase matrix mineralization in the case of hFOB 1.19 cells. Our results are in accordance with results reported in other publications, where various NCD coatings (including O-NCD) promoted increased ALP activity in SAOS-2 osteoblastic cells in comparison with a reference polystyrene culture dish^27,28^. Moreover, other researchers also did not observe increased expression of collagen type I, osteocalcin or osteopontin in SAOS-2 or MG-63 osteoblastic cells cultured on O-NCD samples^28,74^, whereas neonatal human dermal fibroblasts formed an extracellular matrix with collagen both on O-NCD and on H-NCD^75^. Matrix mineralization or other osteogenic markers investigated in our study were not evaluated in the study by Kalbacova et al.^27^. In contrast to our results, Liskova et al*.*^28^ reported increased matrix mineralization in the case of SAOS-2 cells grown on O-NCD coatings. There are various possible explanations for this discrepancy, e.g., the use of different analysing methods, or more likely the use of different reference controls (the polystyrene culture dish vs. the Ti6Al4V alloy) or different cell lines. Unlike non-tumour osteoblast cell line hFOB 1.19, the SAOS-2 cell line is derived from a malignant bone tumour and has various chromosomal abnormalities and gene mutations^60,76^, resulting in different cell behaviour in response to the same culture substrate or in response to the same culture conditions, with SAOS-2 cells expressing the most mature osteoblastic phenotype^77^. The differences between non-tumour and tumour-derived osteoblast cell lines have been thoroughly discussed in a recent publication by Nemcakova et al*.*^78^.

Functionalization of the O-NCD coating with BMP-7 markedly improved the osteogenic maturation of hFOB 1.19 cells in vitro, which was confirmed by the elevation of all osteogenic markers evaluated in the present study. In complete agreement with our results, other studies have also reported improved osteogenic properties of various Ti-based materials with immobilized BMP-7 molecules on their surfaces. Rat primary osteoblasts, human bone marrow mesenchymal stem cells and mouse MC3T3-E1 pre-osteoblasts cultured on these materials showed increased ALP activity, increased osteocalcin and osteopontin expression, as well as increased matrix mineralization in vitro^38,79,80^.

### In vivo tissue response to various implant surfaces

The tissue reaction in the vicinity of an implant is a multifactorial and multistep process. Its morphological appearance is closely related to the character, composition and structure of the implant surface and to the duration of implantation^81,82^. The granulation tissue and the formation of the woven bone around the implant are present soon after implantation – after approximately 3 weeks^81,83^. Remodelling processes characterized by the formation of the mature lamellar bone surrounding the implant are observed after 4 weeks^83,84^.

**Figure 5:**
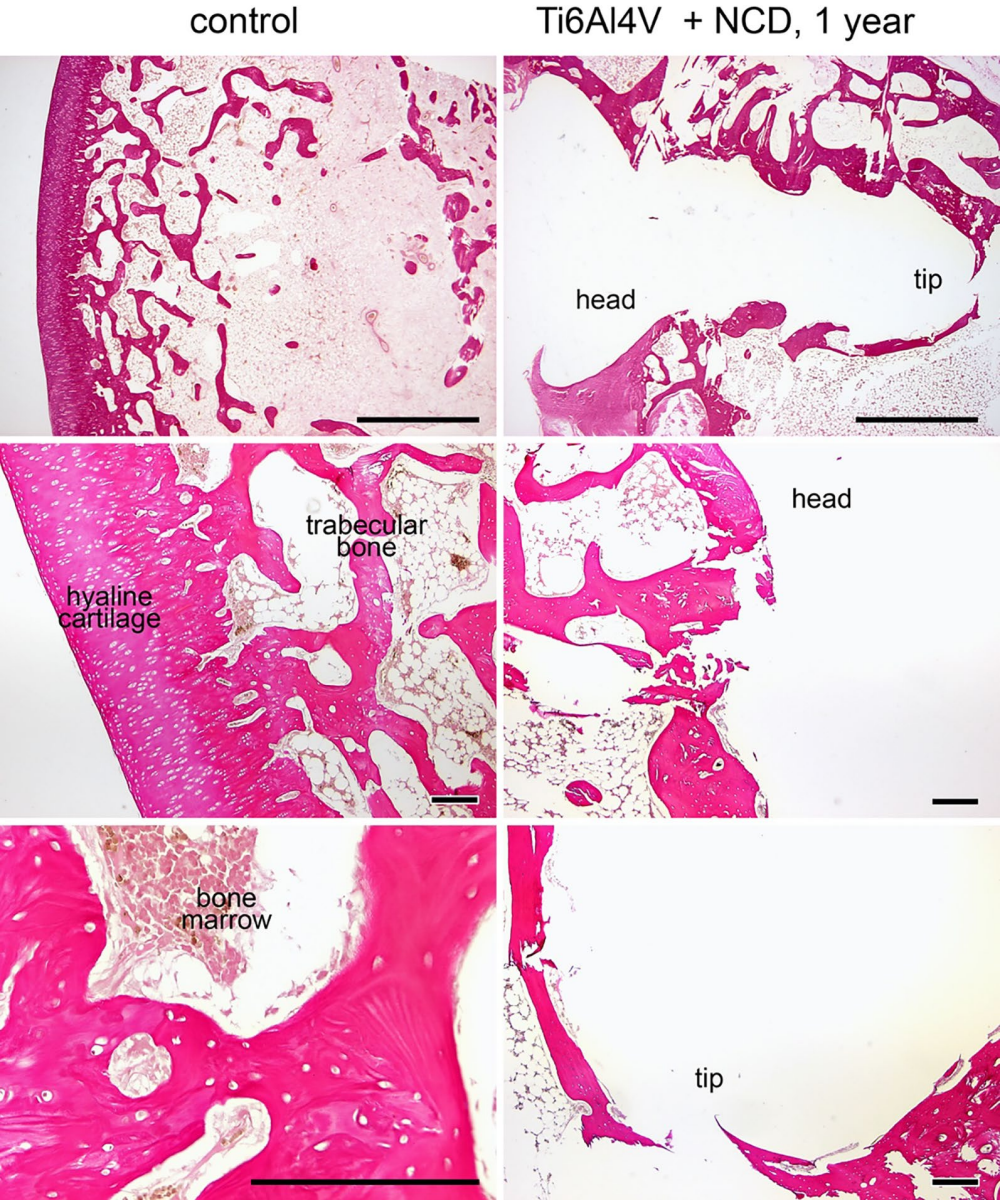
The morphology of distal rabbit femur one year after implantation. Complete embedding of the screw by the bone is visible (top row). The tissue in the contact with the head is partly cartilaginous (middle row), while the tip is covered by newly-formed bone (bottom row). Van Gieson/orcein staining (top row) and hematoxylin–eosin staining, scale bars 1 mm (top row) and 200 µm (remaining panels). Control = unoperated side.

SEM analysis of explanted screws showed over 95% of the surface coating intact (confirmed by element analysis, Ti vs. C, data not shown here), confirming the long-term stability of the NCD coating under in vivo conditions. Our preliminary experiments with a healing period of 6 and 12 months showed excellent osseointegration with a continuous rim formed of trabeculae of lamellar bone (mean thickness 130 µm, range 24–152 µm) in the vicinity of the screw in all groups (Fig. 5) with no differences (both for Ti6Al4V and for stainless steel screws, with and without the O-NCD coating).

**Figure 6:**
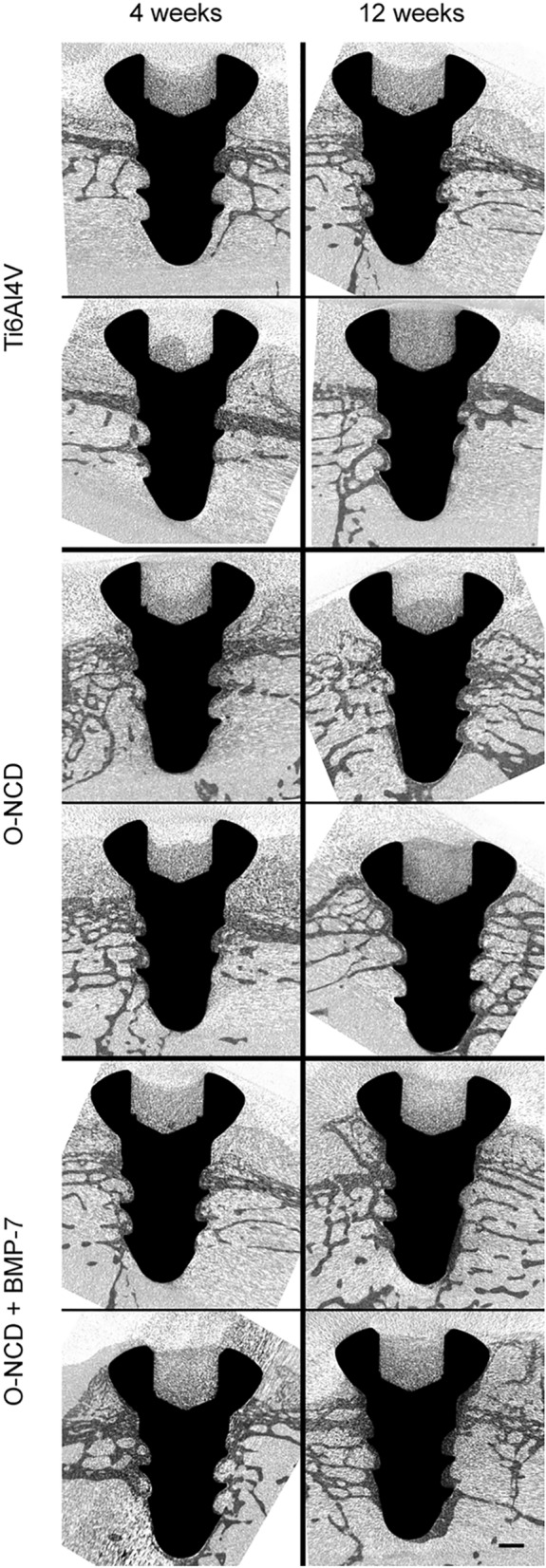
Micro-CT images of implants after 4 and 12 weeks. Two representative cases from each group are shown in longitudinal sections. Increased bone coverage progressing from the screw head towards its tip is evident in all groups. There is clearly more implant coverage in the O-NCD-coated group than in the reference uncoated Ti6Al4V group, and the thickness of the newly-formed bone in contact with the surface of the screw is enhanced in the O-NCD + BMP-7-coated group, especially the tip of the screw. Note also the less mineralized cartilage filling the head of the screw. Scale bar 1 mm.

Further attention was therefore focused on earlier time points (4 and 12 weeks). Three groups of animals (N = 7 per group) received implants first to the right femur, and 8 weeks later received implants from the same investigated group (either the control Ti6Al4V, O-NCD-coated Ti6Al4V, or O-NCD + BMP-7-coated Ti6Al4V) to the left femur, thus reducing the experimental variation and also the numbers of animals in the experiment. After an additional period of 4 weeks, a non-destructive ex vivo whole specimen 2D/3D visualization and analysis was performed by micro-CT. Another advantage of micro-CT 3D visualization is that it may also improve the preparation of subsequent histological sections (i.e., the position and the orientation of the section). The micro-CT results (Fig. 6) showed clear temporal progress in osseointegration. The extent of trabecular bone in contact with the O-NCD-coated implants was clearly and consistently higher than in the bare Ti6Al4V implants. A further increase in the extent of bone formation and in the thickness of the newly-formed bone in direct contact with the implant surface was observed in the O-NCD + BMP-7 implants at both 4-week and 12-week time points.

**Figure 7:**
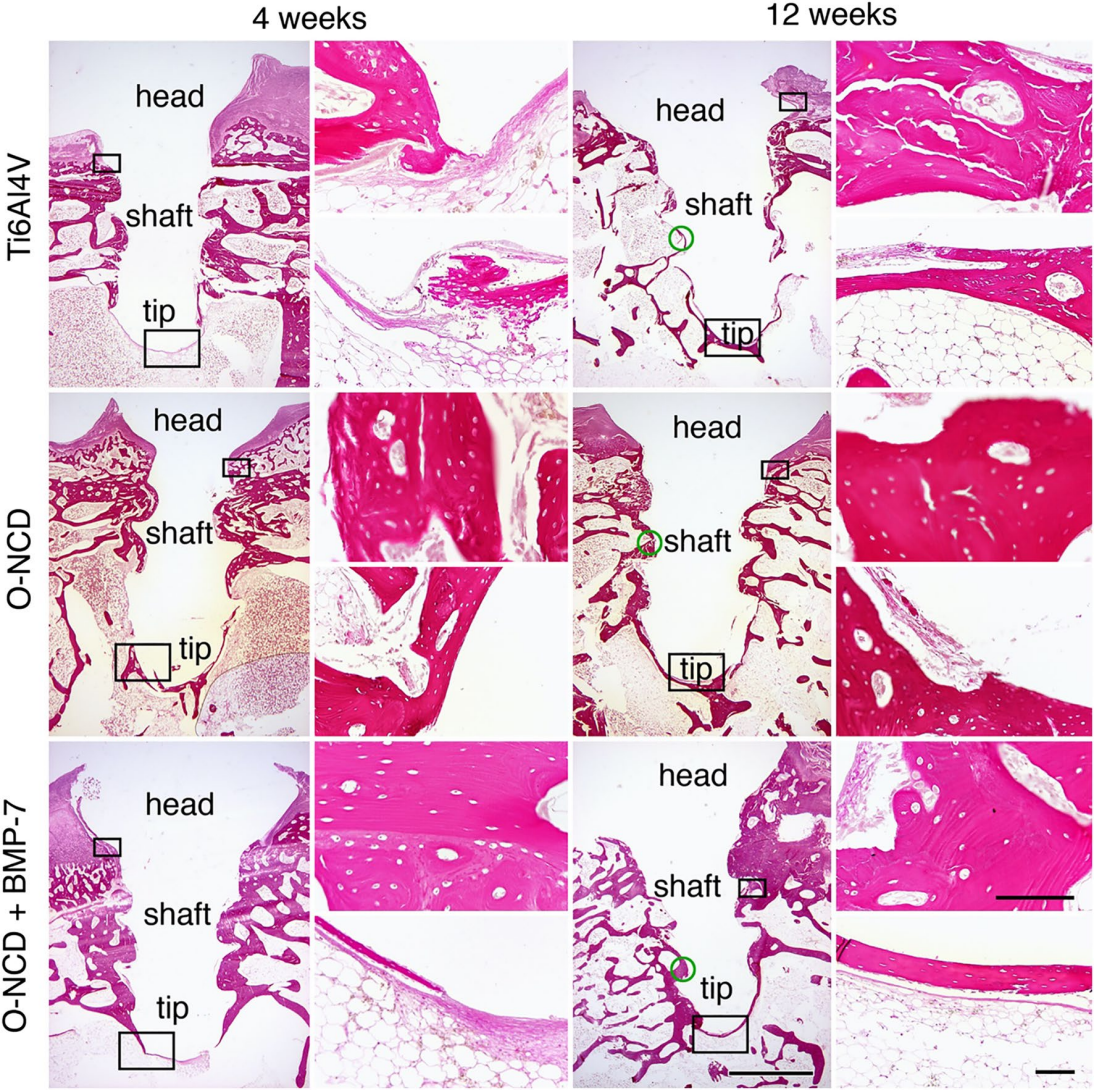
The histology of the implant site 4 and 12 weeks after the procedure. In contrast to bare metal implants, both coatings promoted bone formation at the screw tip at 4 weeks. Increased thickness of the newly-formed bone is evident in the O-NCD-coated group with a further increase in the O-NCD + BMP-7-coated group in both time intervals, confirming the micro-CT data (Fig. 6). The panel on the left shows the whole implant in elastin staining at low magnification, and the panel on the right shows higher power views from the region of the head and the tip (hematoxylin–eosin staining). Empty spaces correspond to fat cells in the bone marrow. Boxes indicate the approximate position of the high-power views taken on the sister sections. Green circles indicate points of measurements for quantification. Scale bars 1 mm (overview images), 100 µm (high power views).

Serial histological sections (Fig. 7) also confirmed the observation from the micro-CT revealing greater thickness of the newly-formed bone on the O-NCD with a further increase on the O-NCD + BMP-7 surface at both time intervals. There was no foreign body reaction on the surface of the coated implants, and we did not observe any coating detachment with loose parts containing nanodiamonds on or in the bone. Similarly to our observations, good agreement between the micro-CT results and the histology results in the evaluation of the bone-implant contact analysis has also been reported in other studies^85–87^. The more detailed histology results (Fig. 7) also showed that the mineralization of the connective tissue progressed from the head of the screw towards the tip. The tissue found around the head was histologically hyaline cartilage (from the joint cartilage), with the cavity in the screw head often filled by newly-formed fibrocartilage, which was also formed in the joint capsule that came into contact with the implant. At 4 weeks, the discontinuous rim of the lamellar bone-forming trabeculae and areas of the bone marrow circumscribed by a thin layer of collagen connective tissue was present around the screw. A thin rim of fibrous tissue continuing to the bone marrow covered the tip of the screw. A downward arrangement of osseointegration of this type has also been observed in other publications^84,88^. At 12 weeks, the continuous rim of the newly-formed lamellar bone was present in most of the implants, including the area of the tip.

**Figure 8:**
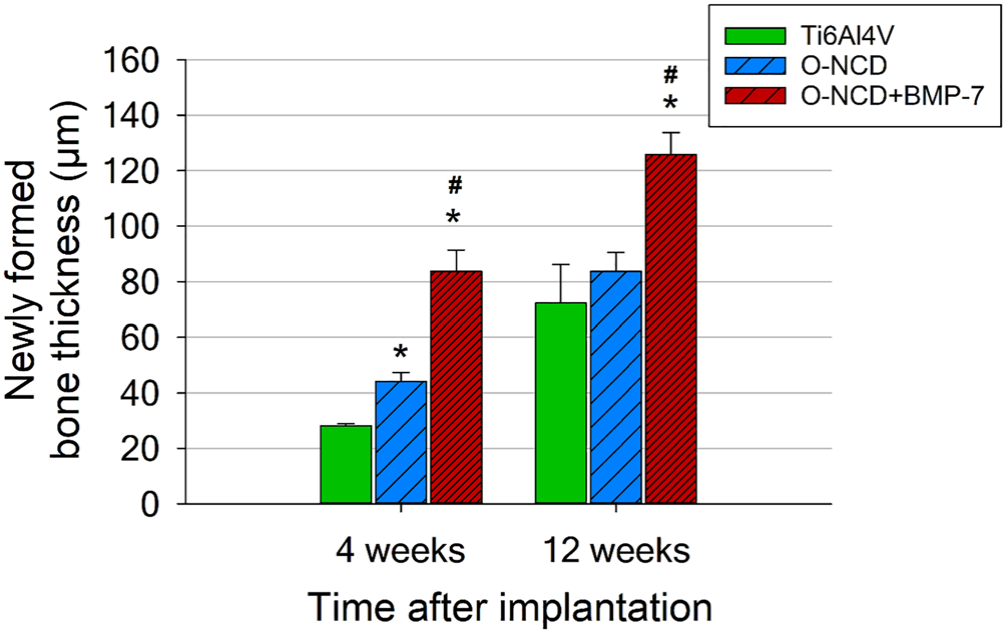
Quantification of newly formed bone at mid-shaft at 4 and 12 weeks after the implantation. Mean ± S.E.M., *p < 0.05 vs. Ti6AL4V, #p < 0.05 vs. O-NCD (unpaired two-tailed t-test).

We quantified the thickness of newly-formed bone in all three experimental groups by measuring, for consistency, the region of the mid-shaft of the screw (illustrated by green circles in Fig. 7). In all groups, the thickness increased between 4 and 12 weeks (paired t-test, p < 0.05, Fig. 8). There was a significant difference between bare metal and coated implants at 4 weeks, while at 12 weeks, the difference was only significant for the O-NCD + BMP-7 group (Fig. 8). At both time points, the layer of the newly-formed bone was significantly thicker in the O-NCD + BMP-7 implants compared to the O-NCD group.

The faster bone formation promoted by the O-NCD coating observed in our study was also reported by Kloss et al. After 4 weeks of implantation in sheep calvarias, O-NCD-coated Ti dental implants showed a thicker layer of mineralized osseous tissue than uncoated Ti implants^30^. By contrast, two other studies investigating the effect of NCD coatings on the osseointegration of Ti6Al4V implants in domestic pigs or in New Zealand white rabbits failed to show any positive effect of NCD coatings^32,33^. Moreover, the NCD-coated implants became osseointegrated at a later time point than the uncoated implants^33^. This discrepancy with our results can be explained by the use of NCD coatings without the post-deposition oxygen plasma treatment that was applied in our study. The as-deposited NCD diamond coatings are generally hydrogen-terminated. A comparison of the wettability of NCD coatings with various post-deposition treatments showed that both the as-deposited NCD coatings and the hydrogenated NCD coatings were less hydrophilic than the oxygen plasma-treated O-NCD coating^89,90^. The hydrophilic O-NCD films improved the adsorption of cell-adhesion-mediating proteins, and showed two times higher biological activity and better cell adhesion, proliferation and osteogenic differentiation in vitro than the H-NCD films^28,90^. An oxidized NCD coating should therefore also better promote osseointegration in vivo than an as-deposited NCD coating or an additionally hydrogenated NCD coating. Indeed, the study by Kloss et al*.* confirms this explanation. While the O-NCD-coated implants promoted new bone formation better than the uncoated implants, the H-NCD-coated implants showed even less bone formation than the uncoated implants. Moreover, although the O-NCD-coated implants were overlaid with a thick layer of mineralized osseous tissue, the H-NCD-coated implants were covered with accumulating fibrous tissue^30^.

The functionalization of O-NCD-coated implants with BMP-7 protein further increased the thickness of the newly-formed bone, as was confirmed by both micro-CT and histological analysis in our study. A recent publication has also shown accelerated new bone formation induced by calcium phosphate + BMP-7-coated Ti implants in goats with osteoporotic-like bones within the first month after implantation^91^. Similar results have been reported in some other publications, where BMP-7-modified Ti or Ta implants promoted faster new bone formation in dog jaws or in rabbit femurs, respectively, than the control metallic implants without BMP-7^44–46^. These results suggest that biological functionalization of bone or dental implants with a strong osteogenic factor such as BMP-7 protein can markedly accelerate the post-surgical healing process. This can be especially important in patients with a hostile bony environment caused by a disease like osteoporosis. However, it is important to mention that the osseointegration process depends on the selected animal model, and this affects the extrapolation of healing dynamics from animal studies to humans. Botticelli et al*.* have reported that bone healing occurred much faster in smaller mammals such as rabbits than in humans^92^.

## Conclusions and further perspectives

Material and morphological analyses have shown the conformal nanocrystalline diamond coating of Ti6Al4V plates and Ti6Al4V screws with a fine nanostructured diamond morphology. AFM confirmed the homogeneous coating and the tight binding of BMP-7 molecules on diamond surfaces. It also revealed the nanostructured morphology of the adsorbed BMP-7 layer, where molecules lie on the surface and form nanoscale aggregates. A combination of in vitro and in vivo data has shown (1) no adverse effect of an oxidized nanocrystalline diamond coating with good long-term stability and tolerance and (2) the possibility of functionalizing this O-NCD coating with BMP-7 protein, resulting in a considerable increase in mineralization of the matrix in vitro and significantly faster osseointegration in vivo. Implants coated by nanocrystalline diamond, especially with further functionalization with bioactive molecules, such as BMP-7, can provide significant added value and medical benefits in clinical applications such as orthopaedic or dental implants (1) by promoting faster osseointegration resulting in better bone healing with the possibility of earlier loading, and (2) by limiting allergic reactions to the metal by shielding it from the immune system, which can also be expected (and which could be an interesting topic for further studies).

## Data Availability

The datasets generated during and/or analysed during the current study are available from the corresponding author on reasonable request.
